# Adult Health and Early Life Adversity: Behind the Curtains of Maternal Care Research

**DOI:** 10.3389/fphys.2022.804239

**Published:** 2022-03-03

**Authors:** Theodore C. Dumas

**Affiliations:** Psychology Department, Cognitive and Behavioral Neuroscience Program, Interdisciplinary Program in Neuroscience, George Mason University, Fairfax, VA, United States

**Keywords:** early life adversity, deprivation, epigenetics, failure to thrive, maternal care, postnatal development, stress

## Abstract

The quality of one’s adult health and the chances of maintaining cognitive ability in aging stem directly from the quality of care one receives as an infant. Formal studies of maternal care can be traced back at least a century. Revelations of behavioral outcomes after maternal deprivation in primates were followed by discoveries of systemic and brain growth factors mediated by the caregiver–offspring relationship in rodents. More recently, much of the genetic/epigenetic bases of maternal care has been defined and positively linked to adult health and cognitive ability in senescence. The history of this field is both tragic and fascinating. The early primate work, while informative, was abusive. The initial rodent work was ridiculed before its importance was recognized. The final lesson learned is that infant/toddler care matters a lot. Today, we have a better understanding of the biology underlying maternal care and its transmission across generations as well as a scientific basis for massaging premature infants and hugging our children.

## Introduction

Altricial animals require parental care during the neonatal period. Nurturing physical and emotional interactions between offspring and caregivers are instrumental in the processes of early postnatal growth and brain maturation (Bergman et al., 2019). Neglected or abused offspring often display developmental disturbances including weight loss, stunted growth, gastrointestinal disorders, impaired brain development and cognitive control, and immune incompetence (Homan, 2016; Wade et al., 2018; Park et al., 2021). Longer term, neglected, or abused offspring display an increased propensity for obesity and a variety of adult diseases (Weaver, 2009; Cagampang et al., 2011; Godoy et al., 2021; Grummitt et al., 2021). These and numerous other conditions arising from maternal deprivation in humans are thought to stem from reduced levels of essential growth factors (Kuhn and Schanberg, 1998) and increased stress hormone release (Rosenfeld et al., 1992; Liu et al., 1997). Those reared without a nurturing caretaker almost invariably become unhealthy and dysfunctional adults. Research into the physical and psychological outcomes of varying types and degrees of parental care can be traced back over a century. The biological bases of the impacts of parental care on offspring growth have been studied for over 50 years. Most recently, key aspects of the epigenetics of parental care have been identified and help to clarify how the effects of early life experiences persist into adulthood and aging and how such effects can be transmitted across generations. This is a situation where nurture wins out over nature, where maternal wisdom dominates, where quality of adult life hinges on intimate physical and psychological bonding during the neonatal period. Greater attention to this phenomenon and application of the findings by parents, care givers, and policymakers would very likely result in a qualitatively healthier society.

## The Plight of Baby Gua (and Donald)

In retrospect, the nature versus nurture debate appears to have started off as an inappropriate zero-sum argument. It was believed that who we become as adults, our mental and physical characteristics and abilities, was either due to genetics or experience, sometimes both. Likewise, early experiments to address the nature/nurture dilemma were simplistic, not well controlled, and for one well-recognized study, in the end, was relatively thoughtless and lacking compassion. During the 1920s, the husband-and-wife team of Luella and Winthrop Kellogg brought home with them from Cuba an infant chimpanzee named Gua, at seven and a half months of age. She was to be reared side-by-side with their son Donald, who was 10 months old at the start of this experiment. The basic question was, is a chimp a chimp because it has chimp genes or because it is raised by other chimpanzees? Cross-fostering separates these two variables. Thus, if baby Gua grew up to be very chimp-like in a human household, then genes win. However, if baby Gua grew up to be more human than chimp, then environment wins. As such, Gua was treated like Donald’s sister and underwent the same bathing, dressing, and feeding processes. They spoke to Gua the same as they did to Donald and played with her in similar ways. The study started out successfully and numerous measures of physical and cognitive development were obtained (Kellogg and Kellogg, 1933). Overall, baby Gua outperformed Donald in terms of gross and fine motor skills. She walked upright earlier and sat unassisted in her highchair earlier. At the same age, Donald had to be strapped into his highchair or risk tumbling out. When applying the same assessment criteria, Gua also learned the meaning of a similar number of words, but she did it 2 months earlier than Donald. Strikingly, Gua acted very much like a human infant in her need for physical contact and emotional reassurance. She required the same parent–infant bond as did Donald, something seemingly overlooked during the planning of this study. The experiment was ended early because the Kelloggs felt Donald was becoming too much like Gua, for example barking upon food presentation. Winthrop Kellogg’s final conclusion was “*Gua, treated as a human child, behaved like a human child except when the structure of her body and brain prevented her. This being shown, the experiment was discontinued*.” (Babe and Ape, 1933).

When the study was terminated, the Kelloggs sent Gua to the Yerkes Primate Center in Florida. This was the second time she was stripped from her “mother/caregiver,” and she went from a warm affectionate family life to a relatively barren cage with other strange and not so well-behaved chimps. She died less than a year later, *circa* her third birthday, of a broken heart (the official cause of death was determined to be pneumonia). In this story, we possibly learn more about the importance of the caretaker–infant bond than we do about nature versus nurture. First, the caretaker–infant bond is real and of life importance (win for nurture). Second, the caretaker–infant bond can occur across species given that Gua thrived with the Kelloggs as replacements for her biological mother (win for nurture). Third, the final outcome of this study clearly illustrates that the primate is not an ideal animal model for this type of research. Fourth, one’s own human infant is not a reasonable subject for scientific research. While direct connections cannot be made to being an infant research subject, being reared with a chimpanzee, or losing his “sister” as a toddler, Donald committed suicide at age 43 (Deese, 1973). Rightly so, this scientific approach would not be approved by any institutional animal care and use committee or institutional review board today.

## Isolating Infant Monkeys Leads to Isolated Adult Monkeys

While Kellogg and Kellogg were publishing their findings, Sigmund Freud was making his big splash in the clinical literature about the infant mother bond and effects of early life trauma on adult mental health (Freud, 1936; Stern et al., 2010). This is also when the famous and perhaps infamous Dr. Harry Harlow entered the story while attempting to identify critical developmental factors in primates. Unlike the Kelloggs, Dr. Harlow performed his investigations in a laboratory with Rhesus monkeys and with far better controlled experiments. In this research, infant monkeys were separated from their mothers the day after they were born and then reared in isolation by two side-by-side surrogate mothers (Harlow, 1958). Both surrogate mothers were little like real moms. They were inanimate. One was made mostly of wire mesh, and the other was covered with terry cloth. Food was available with the wire mesh surrogate, but never with the terry cloth surrogate. Prior to this research, it was simply assumed that the bond between infant and mother is largely dependent on the fact that mom provides rewarding food. The results from this study turned that notion on its head. The isolated infant monkeys spent far more time with the terry cloth surrogate than with the wire mesh surrogate, despite the lack of food provision by the terry cloth surrogate. The terry cloth surrogate provided a substrate for clinging, which served as a comfort mechanism for the separated infants (Seay and Harlow, 1965). Thus, one general finding from Harlow’s research was that infant bonding is based on more than just food and bias toward the terry cloth surrogate suggested something tactile. However, terry cloth does not abrogate the loss of one’s mother. As adults, all of the monkeys isolated as infants were socially maladjusted spending most of their time alone or fighting with other monkeys. Those females isolated as infants became careless, brutal, and rejecting caretakers for their offspring. These behavioral outcomes and persistence of effects into adulthood provided a partial explanation for cross-generational effects of maternal deprivation. Inadequately reared infants become inadequate providers as adults, and so on.

The findings from Harlow’s studies rocked the scientific world. However, practical application for the benefit of humans did not immediately follow. At the same time that Harlow’s work was being published, orphaned children in eastern Europe were being housed individually, with little human contact. “Neglect” is a conservative term applied to this situation. These unfortunate children were locked in their rooms in isolation, interacting with other humans only briefly during feedings. Given the results from Harlow’s studies, the outcome for these orphans could be predicted. Many did not survive their ordeal in the orphanages. Of those who did survive and were adopted, features, such as stunted growth, reduced brain size, cognitive impairment, delayed language acquisition, severe anxiety, depression, and antisocial behavior, were prevalent (Bucharest Early Intervention Project). Most of those who survived into adulthood exhibited enduring physical and psychological debilitations.

By the late twentieth century, decades of research had confirmed that maternal deprivation is inhumane for humans, apes, and monkeys. Nonetheless, Dr. Ned Kalin at the University of Wisconsin earned a grant from the National Institutes of Health in 2014 to perform similar research. Dr. Kalin had been studying stress responsiveness in maternally separated monkeys since the late 1980s. A concerted effort by Mr. Rick Bogle, former Executive Director of Alliance for Animals, a petition signed by hundreds of thousands of concerned citizens, and likely action by the University of Wisconsin Board of Visitors in 2015, resulted in removal of the maternal deprivation portions of Dr. Kalin’s research. Dr. Kalin is an MD with a focus on treating human diseases. As the medical system in the U.S. continues to shift from treatment to prophylaxis, so too will the underlying basic and translational research shift from primarily studying pathology to a greater concentration on wellbeing and from deprivation to enrichment.

## The Golden Goose Award

What did subsequent rodent studies tell us about maternal deprivation? In the 1970s, Dr. Cynthia Kuhn and Dr. Saul Schanberg then at Duke University set out to discover the biological factors that were critical for infant growth and quickly found that nutrient sustenance was simply not enough. They isolated rat pups from their mothers and fed them, but the pups did not grow (KuhnÄ et al., 1978). Under the skin, they had reduced systemic levels of growth hormone and less ornithine decarboxylase (ODC) in their brains (KuhnÄ et al., 1978), a translator for the effects of growth hormone on soft tissue and bone. Corticosterone (stress hormone) levels were unaltered, so the problems associated with maternal deprivation in the early neonatal period were not due to gross hypothalamic or pituitary breakdown. Instead, the effects of were specific to certain hormonal systems. *Sidenote: similar procedures imposed on older rat pups do alter corticosterone levels and disparate types of stressors do not elevate corticosterone in very young rats* (Rosenfeld et al., 1992). *This resulted in the first 2 weeks of postnatal development in rats being defined as a stress hyporesponsive period* (Ganella and Kim, 2014). This partially answered the growth attenuation dilemma but did not address the cause of the biological effects of isolation from the dam. So, they reinstated individual characteristics of the dam to different groups of isolated pups (Evoniuk et al., 1979). Some pups could smell the dam, but could not see, hear or touch her. Others could hear the dam, but could not smell, see or touch her. Regardless of the condition, these isolated pups still had reduced ODC levels and reduced growth. Even reintroduction of an anesthetized dam was insufficient to reinstate growth, though the pups fed from her teats. The story was very different when an awake dam was reunited with the pups. She would dig out a nest in the corner of the cage, drag all of the pups into the nest, and then lick and groom them incessantly. This licking and grooming reinstated normal growth. In fact, growth hormone and ODC levels in the brains of pups separated and then returned to the dam exceeded those found in control animals that were not separated from the dam, though the reason was not known at the time (KuhnÄ et al., 1978).

In general, maternal behavior is highly complex involving numerous actions on the part of the dam and offspring (Champagne et al., 2003) and is differentially modified by different forms of maternal stress (Murgatroyd and Nephew, 2013; Orso et al., 2019; Rajasekera and Gur, 2021). Studying the effects of stress on maternal behavior is important because roughly 10–20 % of human mothers suffer postpartum depression (American Psychological Association), which impedes maternal care and development of her offspring (Lupien et al., 2000; Nelson et al., 2018). Gary Evoniuk, in collaboration with Kuhn and Schanberg, carefully watched the moms that were reunited with the isolated pups. They were not gentle. They were rough in their attention. Cleverly, Dr., Evoniuk took a camera lens brush and applied 10 vigorous strokes to the back of the head/neck of each experimental pup every 5 min (Evoniuk et al., 1979). This manipulation reinstated growth in the pups. Thus, among the orchestra of natural maternal behaviors expressed postpartum, there was something special about this tactile stimulation in the process of infant/mother bonding and for the pups to thrive (Schanberg et al., 1984; Pauk et al., 1986).

The importance of maternal care on adult responsiveness to stress was reinforced by the work of Seymour “Gig” Levine and colleagues. Dr. Levine was heavily influenced by Freud’s infant–mother reports and was eager to understand the biological outcomes of early life trauma. After examination of the mother–infant relationship in monkeys, Dr. Levine turned to the rat model. During the primary studies, rat pups were briefly separated from the dam and received moderate electrical shocks (Levine, 1956; Levine et al., 1956). One control group was handled the same as the shock group and even placed in the shock chamber but did not receive any electrical shock. A second control group was not handled at all. When the three groups were tested in an avoidance learning task as adults, unexpectedly, the poorer performance of the unhandled group stood out. The animals that were shocked or handled as newborns showed greater resilience and more adaptive behavioral responses as adults. To some degree, this initial report countered the findings of Kuhn and Schanberg and prompted the creation of an entire field studying early handling/infantile stimulation and the benefits for coping with stress in adulthood. However, continued research identified maternal care as a key mediator of the adult outcome for the handled pups. When the handling or shock process was completed and the pups were returned to their respective dams, the dam treated the pups from the three experimental groups differently. Handled and shocked animals were retrieved more quickly, nursed more, and received far more attention and licking and grooming than the unhandled controls (Thoman and Levine, 1970; Smotherman et al., 1977). Thus, the enhanced ability to deal with stress in adulthood in the shocked and handled animals was a function of greater maternal care. These results clearly illuminate the power of maternal care to overcome early life adversity.

Tiffany Field, a developmental psychologist at the University of Miami School of Medicine, became aware of the isolated rat and tactile recovery experiments and joined Kuhn, Schanberg and Evoniuk to extend the research to the human level. Fifteen min of human massage to stable premature infants in incubators for as few as 10 days resulted in a nearly 50% increase in growth rate compared to premature infants who were not massaged (Field et al., 1986; Dieter et al., 2003). The massaged premies also displayed longer wake times (Dieter et al., 2003) and reduced stress behaviors (crying, grimacing, jerking, startle; Hernandez-Reif et al., 2007). Subsequent longitudinal studies revealed reduced hospital stays (Badr et al., 2015) as well as reduced stress hormone levels and increased immune competence in the massage group (Badr et al., 2015). Dr. Field’s own daughter was born prematurely and served as a “guinea pig” for this study. Dr. Fields’s testament to the success of this case study is that her daughter is taller and smarter than she is.^1^ Overall, this research has led to the more general practice of massaging premature infants and has resulted in earlier removal from the incubator and improved physical and cognitive outcomes for the babies. Equally important, nearly every hospital in the modern world now applies the “skin-to-skin” approach for newborns where, after some cleaning and collection of vital measures, the newborn is placed naked on the bare mother’s chest and they are both covered with a soft light blanket, typically for over an hour. Newborns who undergo this skin-to-skin practice lose less weight postpartum, grow faster, thermoregulate better, and latch on and begin to suckle earlier than newborns who do not experience skin-to-skin (Gupta et al., 2021), and there are lasting positive effects on the mother–child relationship (Bigelow and Power, 2020). Thus, maternal care not only stimulates the release of growth hormones and factors that enhance newborn health, physical contact between caretaker and offspring immediately after birth promotes behaviors in the offspring that optimize its growth.

By the start of the 21st century, it had become crystal clear that caregiver–infant attachment is an indispensable part of normal physical, emotional, and cognitive development in most mammals, including humans. Regardless, beginning in 1975, Senator William Proxmire of Wisconsin regularly issued what he called “Golden Fleece Awards” mocking federally funded research he thought was odd or a misuse of federal money. Massaging baby rats was not spared from criticism. At the end of the 1980s, Representative Jim Cooper of Tennessee reversed the legacy of the Golden Fleece Award by establishing the “The Golden Goose Award” to raise awareness of the misunderstanding of how science works and how groundbreaking discoveries (Golden Eggs) come to fruition from federal funding (the Golden Goose). Now conferred annually by the American Association for the Advancement of Science, the Golden Goose Award is presented to researchers who, through federal funds, made findings that significantly altered society or medicine, but upon initial discovery, were considered relatively inconsequential or impractical by other researchers and politicians. Cynthia Kuhn and Saul Schanberg, Gary Evoniuk, and Tiffany Field received this award in 2014 for their work on maternal separation and the importance of neonatal massage and tactile stimulation (see Footnote 1). An award well-deserved.

## Nature *via* Nurture

To this point, much had been gleaned about the behavioral and biological outcomes of maternal deprivation, but one big question remained. How does mom’s behavior, or lack thereof, create the lasting effects in the offspring that persist into adulthood? Michael Meaney at McGill University was keen on this issue and, like Dr. Evoniuk, paid close attention to the dam-pup interactions. He noticed substantial variability in the ways the dams treated their newborns. Really good moms dug a nest in the corner of the cage, dragged all the pups there, created a tent with her body over the pups (arched-back nursing), and licked and groomed them at a very high rate. In contrast, bad moms dug a nest and laid down. The pups had to approach the bad mom on their own and periodically she would lick one or another pup. The behavior of most dams was somewhere in between these two extremes. So, Dr. Meaney along with Dr. Paul Plotsky, then at the Salk Institute, quantified licking and grooming rates, calculated the mean across the dams, rank-ordered the dams according to licking and grooming rates, and defined those above the mean as “high-rate” licker-groomer dams and the rest as “low-rate” licker-groomer dams (Liu et al., 1997). Like humans, rats will adopt offspring that are not their own and rear them as their own. So, they took a couple of pups from one litter and added them to a litter with a high-rate mom and another couple of pups from the same origin litter and added them to a litter with a low-rate mom. This litter splitting procedure ensured balanced genotype effects in both rearing groups without altering the behavior of the adopting dam, which is sensitive to stress and manipulation of the offspring (Orso et al., 2019). The outcome for the pups was very clear. In adulthood, those pups reared by the high-rate dams displayed blunted stress responses, reduced corticotropic releasing factor in the hypothalamus and increased expression of glucocorticoid receptors in the hippocampus (Liu et al., 1997) as well as reduced circulating adrenocorticotropin releasing hormone (ACTH) when compared to the pups reared by the low-rate dams (Liu et al., 2000). Increased numbers of glucocorticoid receptors in the hippocampus yielded greater reactivity to circulating corticosterone levels and greater feedback inhibition of the stress response. Early life nurturing, more than genes, programmed stress responsivity in adulthood.

The effects of maternal care were not limited to the hormonal stress response. GABA_A_ receptor levels in the locus coeruleus, nucleus of the tractus solitarius, amygdala and frontal cortex (Caldji et al., 2000), and vagus nerve activity were all reduced in maternally deprived pups, which led to increase fear responding and anxiety levels in adulthood. Concurrently, reductions in NEp autoreceptors at NEp synapses in the locus coeruleus (Liu et al., 2000) resulted in increased NEp release due to restraint stress in maternally deprived pups. Due to some or all of these factors, females reared by low-rate licking and grooming dams themselves became low-rate licking and grooming dams (Francis et al., 1999), providing some biological bases to Harlow’s earlier observation of transgenerational effects in primates. Meaney and Plotsky, along with Myron Hofer (Hofer, 1981, 1996), proposed that the programming of the stress response early in life *via* maternal care strongly influenced the likelihood of the development of stress-related psychological disorders later in life. In fact, in collaboration with Robert Sapolsky at Stanford University, Meaney et al. showed that high-quality early life care promoted successful cognitive aging by staving off a degenerative cascade of events in neurons resulting from a lifetime of excessive glucocorticoid release (Meaney et al., 1988). Since then, aging outcomes have been tied to the epigenetics of early life stress (Szyf, 2021). Moreover, the epigenetics of aging are shown to be altered in the offspring of mothers who themselves experienced childhood trauma (Nwanaji-Enwerem et al., 2021), solidifying a biological basis for transgenerational effects on the aging process. This lifespan approach to understanding maternal care and early life adversity has been validated numerous times and extended to circadian clocks, including the diurnal cortisol rhythm (Cagampang et al., 2011), and systemic diseases, such as cardiovascular disease, diabetes, and metabolic syndrome (Weaver, 2009; Seal and Turner, 2021).

Over beer one evening while attending a scientific meeting in Barcelona, Dr. Meaney discussed these maternal behavior findings with a skeptical Dr. Moshe Szyf, a pharmacologist at McGill. At the time, Dr. Szyf had been working on understanding the role of DNA methylase activity, particularly in the context of cancer. Perhaps it was the beer, but Dr. Szyf agreed to collaborate with Dr. Meaney to penetrate the neuronal nucleus and take a look at how the changes in CRF and glucocorticoid receptor levels came about. What they found was remarkable and sparked a flood of research into neuronal epigenetics. It turned out that the pups from low-rate licking and grooming dams displayed increased methylation of the promoter region for the neuronal glucocorticoid receptor gene (Weaver et al., 2004). Greater methylation of the promoter led to reduced expression and reduced number of glucocorticoid receptors in the hippocampus resulting in reduced feedback inhibition of the stress response. Conversely, pups from the high-rate licking and grooming dams had reduced methylation of the glucocorticoid receptor promoter, which resulted in a greater number of glucocorticoid receptors expressed in hippocampal neurons and better regulation of the stress response. Meaney and Szyf eventually showed that the expression levels of roughly 900 genes (about 4% of the total rat genome) were affected by the degree of maternal care (Weaver et al., 2006). Moreover, the methylation state and anxiety outcomes were shown to be reversible in adulthood through pharmacological manipulation of histone acetylation (Weaver et al., 2006) or environmental enrichment (Francis et al., 2002), which might help explain the effects of human therapeutics shown to improve health in aging after early life adversity, such as mindfulness therapy (Sun et al., 2022) and physical exercise (Donofry et al., 2021). Interestingly, therapies directed at the mother, such as interpersonal psychotherapy (O’Hara et al., 2000), cognitive behavioral therapy (Urizar and Muñoz, 2011; McCarthy et al., 2021), mindfulness therapy (Vieten and Astin, 2008), peer support (McCarthy et al., 2021), massage therapy (Onozawa et al., 2001), and maternal–child interaction guidance (Muzik et al., 2015; Fisher et al., 2016), can also be effective in improving parenting and the developmental trajectory of the offspring (Letourneau et al., 2017). Among these, cognitive therapies have been shown to alter functional connectivity in the brain in association with better attention and response to infant cues (Kim, 2021). It is thought that resiliency attributes, such as social support, self-efficacy, and self-esteem, optimize parental capacity and can protect against postpartum stress (Van Haeken et al., 2020). More complete investigation of offspring epigenetic profiles in relation to successful maternal interventions and personal attributes is warranted (Rasmussen et al., 2021).

**Figure 1:**
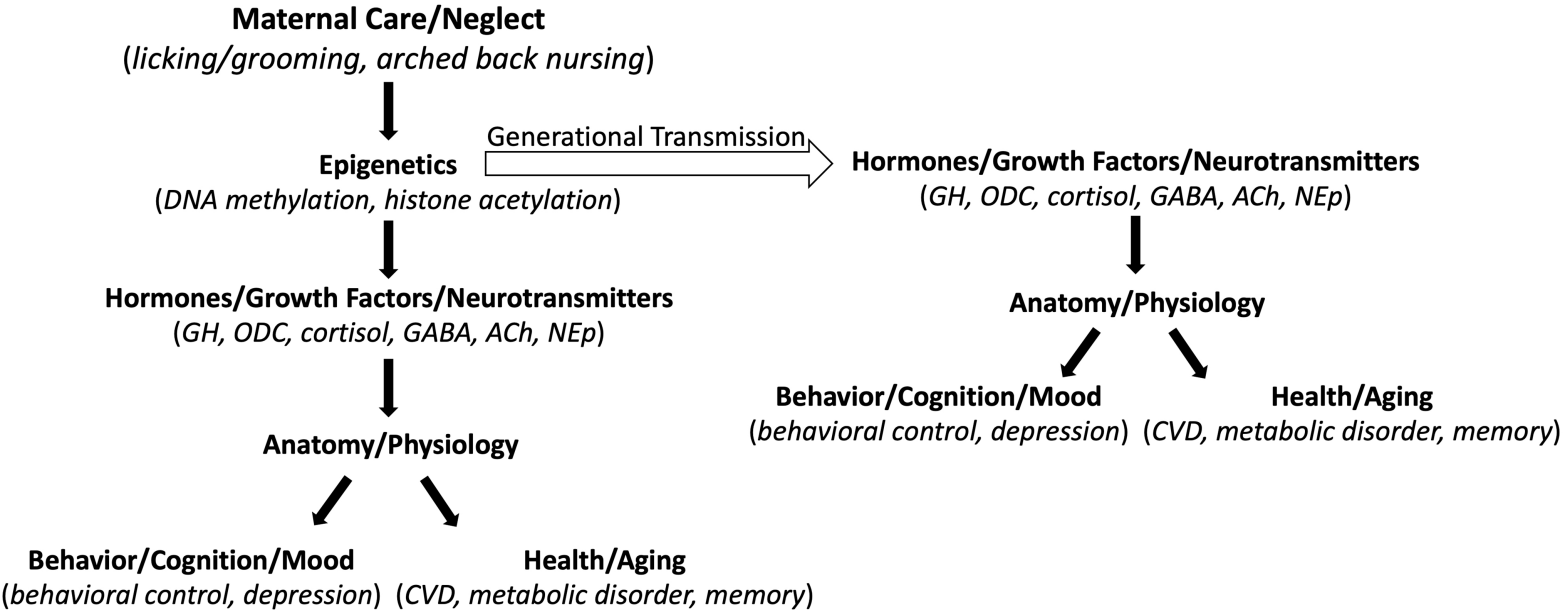
Schematic of the downstream effects of maternal care. Maternal care alters gene expression (Epigenetics) in the offspring that then alters expression of various molecules (Hormones/Growth Factors/Neurotransmitters) that impact quality of life (Behavior/Cognition/Mood) and longevity (Health/Aging). The Epigenetic effects can be transmitted across generations to alter phenotypes in subsequent generations. GH, growth hormone; ODC, ornithine decarboxylase; GABA, gamma-aminobutyric acid; ACh, acetylcholine; NEp, norepinephrine; CVD, cardiovascular disease.

Is this epigenetic process of stress regulation just a rodent thing or does it also happen at the human level? Perhaps the most profound study to date was published by McGowan and colleagues out of the Meaney laboratory in 2009 that made biological comparisons between people who died by accident (mostly automobile accidents) and two groups of suicide victims, those physically abused early in life and those who were not (McGowan et al., 2009). Only the suicide-early abuse group displayed lower numbers of glucocorticoid receptors in the hippocampus and increase methylation of the neuronal glucocorticoid receptor gene promoter region relative to controls. These findings specified early abuse, and not suicidal tendency itself, as the mediator of the alterations in glucocorticoid receptor expression. Direct comparison of the methylation differences between abused and non-abused human children to the methylation differences in rats reared by high-rate and low-rate licking and grooming dams revealed an epigenetic process that is analogous across species (Dunn et al., 2019; Figure 1). What happens in the rodent brain in response to early life stress, down to the molecular level, also occurs in the human brain. More generally, nature–nurture is not a zero-sum game. How we live our lives impacts the expression of given genes in every cell in our bodies. In return, the pattern of gene expression in every cell in our bodies influences how we live our lives. This beautifully complex genetic regulatory process was very well articulated almost 20 years ago in *Nature via Nurture*, by Matt Ridley (Ridley, 2003).

The core work of Golden Goose team, Drs. Meaney and Szyf, and colleagues has been expanded considerably by others to include alterations in gut microbiota in pups experiencing maternal deprivation (Ghia et al., 2008) and attenuation of maternal deprivation induced gut and brain alterations in adulthood after probiotic treatment (Gareau et al., 2007; Desbonnet et al., 2010). Gender differences in the effects of maternal deprivation on susceptibility to depression (Mourlon et al., 2010) and pain have also been identified (Ströher et al., 2019). This story is not complete. Future research will undoubtedly answer remaining questions like, how maternal care is translated into highly specific epigenetic alterations and how the pathological effects of maternal deprivation that persist into adulthood at the human level can be better treated or erased. At present, we might take a moment to marvel at how scientific investigation can be applied to understand what is perhaps the most primal social interaction among humans, the caretaker–infant bonding and rearing process, and its implications for adult mental and physical health.

## Conclusion

Increased public awareness of the importance of the neonate–caretaker bonding and interactions for adult health and greater translation of scientific findings into human practice has the potential to substantially improve health, longevity, and quality of life for perhaps tens to hundreds of millions of individuals. Additional federal funding and larger investments at the state level to support Birth to Three Programs would go a long way in achieving this goal and, in the long term, the increased worker production and reduced healthcare costs would far outweigh the initial investment.^2^

## Author Contributions

All authors listed have made a substantial, direct, and intellectual contribution to the work and approved it for publication.

## Funding

Production of the article was supported by funding from the National Institutes of Health (NIA, 1R15AG060461-01).

## Conflict of Interest

The author declares that the research was conducted in the absence of any commercial or financial relationships that could be construed as a potential conflict of interest.

## Publisher’s Note

All claims expressed in this article are solely those of the authors and do not necessarily represent those of their affiliated organizations, or those of the publisher, the editors and the reviewers. Any product that may be evaluated in this article, or claim that may be made by its manufacturer, is not guaranteed or endorsed by the publisher.

## Footnotes

^1^ https://www.goldengooseaward.org/video/

^2^ https://www.apa.org/pi/women/resources/reports/postpartum-depression
